# SisLeish: A multi-country standardized information system to monitor the status of Leishmaniasis in the Americas

**DOI:** 10.1371/journal.pntd.0005868

**Published:** 2017-09-05

**Authors:** Ana N. S. Maia-Elkhoury, Samantha Y. O. B. Valadas, Lia Puppim-Buzanovsky, Felipe Rocha, Manuel J. Sanchez-Vazquez

**Affiliations:** 1 Communicable Diseases and Health Analysis (CHA), VT, Pan American Health Organization (PAHO), Duque de Caxias, Rio de Janeiro, Brazil; 2 Communicable Diseases and Health Analysis (CHA), Panaftosa, Pan American Health Organization (PAHO), Duque de Caxias, Rio de Janeiro, Brazil; Universidad Peruana Cayetano Heredia, PERU

## Abstract

**Background:**

In the Americas, leishmaniasis is endemic in 18 countries, and from 2001 through 2015, 17 countries reported 843,931 cases of cutaneous and mucocutaneous leishmaniasis, and 12 countries reported 52,176 cases of visceral leishmaniasis. A Regional Information System (SisLeish) was created in order to provide knowledge of the distribution and tendency of this disease to analyze and monitor the leishmaniasis status. This article analyses the performance and progress of SisLeish from 2012–2015.

**Methodology:**

The performance of SisLeish was evaluated by country adhesion, data completeness and delay in entering the data, and also by the SWOT technique. Furthermore, we outlined the structure and *modus operandi* of the system and indicators utilized.

**Results:**

In 2012, only 18% of the countries entered the data in SisLeish before the deadline, where 66.7% and 50% of the countries with autochthonous CL/ML and VL reported their cases to the system, respectively. Whereas in 2015, 59% of the countries reached the deadline, where 94.4% and 58.3% of the countries reported their CL/ML and VL data, respectively. Regarding data completeness, there was great progress for different variables since its launch, such as gender, which had an approximately 100% improvement from 2012 to 2015. The SWOT analysis of SisLeish showed 12 strengths, 11 opportunities, seven weaknesses and six threats.

**Conclusions:**

From 2012–2015 there has been an improvement in the adhesion, quality and data completeness, showing the effort of the majority of the countries to enhance their national database. The SWOT analysis demonstrated that strengths and opportunities exceed weaknesses and threats; however, it highlighted the system frailties and challenges that need to be addressed. Furthermore, it has stimulated several National Programs to advance their surveillance system. Therefore, SisLeish has become an essential tool to prioritize areas, assist in decision-making processes, and to guide surveillance and control actions.

## Author summary

Leishmaniasis is among the most neglected diseases and remains a challenge for surveillance and control programs. In response to this challenge, the Regional Program for Leishmaniasis (PAHO/WHO), with the support of advisors and representatives from endemic countries, have created a Regional Information System (SisLeish), based on epidemiological and operational indicators, to provide knowledge of the distribution and tendency of this disease to analyze and monitor the leishmaniasis status in the Americas. Since the implementation of SisLeish, **t**here has been an improvement in the adhesion, quality and data completeness and the results show the effort of the majority of the countries to improve their national database in terms of completeness and availability. Furthermore, it has stimulated several programs to advance their surveillance system and program, and has been an alternative for countries that currently do not have a national information system available. SisLeish has become an essential tool to prioritize areas, where the occurrence of leishmaniasis requires additional attention, assist in the decision-making process, and to guide surveillance and control actions. This article analyses the performance and progress of SisLeish from 2012–2015.

## Introduction

Leishmaniasis is an infectious disease caused by protozoan parasites of the genus *Leishmania* from the Trypanosomatidae family [1]. It is a vector borne disease transmitted by sandflies from the Psychodidae family, and the genus *Lutzomyia* is the most important vector in the Americas [2]. Leishmaniasis is among the most neglected diseases and are of great importance to public health [2, 3], and due to its epidemiological and clinical diversity, the burden of this disease remains a challenge for surveillance and control programs. Despite having global estimates available, the accuracy of these measures depend directly on the reliability of data collection, which is a result from the strengthening of national surveillance systems [1, 4–7].

Since mid-2000, the World Health Organization (WHO) has worked intensely to integrate leishmaniasis into the political agenda. In 2007, during the World Health Assembly, Member States adopted the Resolution WHA 60.13, assuming the commitment to strengthen the surveillance and control of leishmaniasis, which was reaffirmed by the approval of the Pan-American Health Organization (PAHO)/WHO Resolution, CD 49–19. During a meeting between the PAHO/WHO and representatives from endemic countries of the Americas, in 2008, the participants suggested the development of a regional tool to collect epidemiological data of leishmaniasis, which would standardize, update and release the information to all countries. In 2011, the individual Regional Program for Leishmaniasis (RPL) was instituted by the PAHO/WHO, with the main goals of improving the surveillance and control actions in the Americas, and supporting endemic countries through cooperation, technical consultations and mechanisms to strengthen these actions.

The RPL began defining the epidemiological variables and operation indicators to monitor this disease in the region, in 2012, along with advisors of the PAHO’s communicable diseases, representatives of Leishmaniasis National Programs, and Surveillance Services from endemic countries. In sequence, the RPL developed the Leishmaniasis Regional Information System (SisLeish), which was validated and refined at different stages by the National Programs. The official system was presented during the Leishmaniasis Regional Meeting, held in Panama City on October, 2013, embodying efforts from all endemic countries of the Americas. SisLeish is a simple tool for analysing and monitoring leishmaniasis in the Americas. The system is based on epidemiological and operational indicators, allowing knowledge of distribution and tendency of this disease in the region.

In the Americas, leishmaniasis is endemic in 18 countries, and from 2001 through 2015, 17 countries reported 843,931 cases of cutaneous (CL) and mucocutaneous leishmaniasis (MCL), and 12 countries reported 52,176 cases of visceral leishmaniasis (VL) [8]. In these endemic countries, these diseases are of compulsory notification, whether individual or collective. In some countries, data on the occurrence of leishmaniasis is collected as part of the National Disease Surveillance System, while in others countries there are specific tools for data collection. This information is usually available up to the second sub-national administrative level; however, in some countries this data is only available at the first sub-national administrative level.

Health indicators are the synthesis of measures, which contain information about distinct attributes and dimensions, as well the performance of a health system, and they must reflect the sanitary status of a population and be suitable for surveillance of health conditions [9]. Therefore, the availability of indicators provides inputs for analysis, monitoring of health objectives, promotes professional analytical capacity and the development of information systems. Thus, indicators for disease occurrence are used by governments and international organizations as a tool for decision making, to manage resources and implement strategies, among others [10, 11].

Raw single indicators are widely used in traditional epidemiology to compare situations across different countries and regions, as these are often readily available in most countries and are easy to interpret and to compare [11, 12]. On the other hand, many disciplines prefer composite indicators [13], as they may have a greater contribution to the decision making than the use of single indicators. A previous study of CL showed the limitations of the use of single indicators for epidemiological analyses and defined the high-risk areas to prioritize and plan actions accordingly [14]. The development of an indicator is a process where the complexity may vary from a simple case count of a given disease to more complex methods, such as proportions, reasons, rates or index.

The objective of this article is to analyse the performance and progress of SisLeish from 2012 to 2015. Additionally, this article provides a deep overview of the structure and *modus operandi* of the system and the indicators utilized. This study is intended as a reference to inform current or potential stakeholders involved with leishmaniasis to promote awareness and use of this initiative.

## Material and methods

### SisLeish

SisLeish is an on-line health information system, available in Spanish, developed with the purpose of being a simple tool and user-friendly system for inclusion and consolidation of data, and analysis of leishmaniasis in the Americas. The same provides knowledge and allows monitoring through information from epidemiological and operational indicators of distribution and tendency in the region.

#### Data source

All data available in SisLeish are provided by the Leishmaniasis National Programs or their equivalents from each country that report cases of leishmaniasis, in the Americas. People with access to SisLeish are users appointed by the Ministry of Health (MoH), who are responsible for entering annual information regarding leishmaniasis occurrence and population.

The users enter their annual aggregated leishmaniasis data at the second administrative sub-national level. Moreover, the endemic State Members of the region have agreed upon an annual deadline to enter all of the leishmaniasis data, which is April 30^th^ of each year.

#### System structure

SisLeish is a dynamic web page system created in ASP.NET with the Microsoft SQL Database Server Engine and the Crystal Report to generate data reports and information. This system is structured across three modules: i) module for epidemiological information, such as demographic data, clinical forms, confirmed cases of leishmaniasis by diagnostic criteria and progression of the disease (recovery or death); ii) module for surveillance, control and assistance; iii) specific module for species of *Leishmania* and suspected/involved vectors in the transmission, by region or country. Additionally, the output information is displayed in form of reports and is available by region, sub-region, and country at the first and second sub-national administrative level.

#### Data flow

The users have a specific login to access SisLeish and are responsible for entering the leishmaniasis, population and surveillance, control and assistance data collected annually in their country (each year, until April 30^th^). Countries that have their own information system may fill the data into a standard issued table and import this table into the system; this process is currently being done by only two countries (Brazil and Colombia). Countries that do not have an information system are required to enter manually the data into the system through web forms. Afterwards, the data is imported into the database of the system and the indicators are calculated, generating different outputs, such as demographic, clinical form, diagnosis, treatment, and progression reports, at the first and second sub-national administrative level. These outputs are analyzed and used for epidemiological reports and mapping of the data.

**Fig 1:**
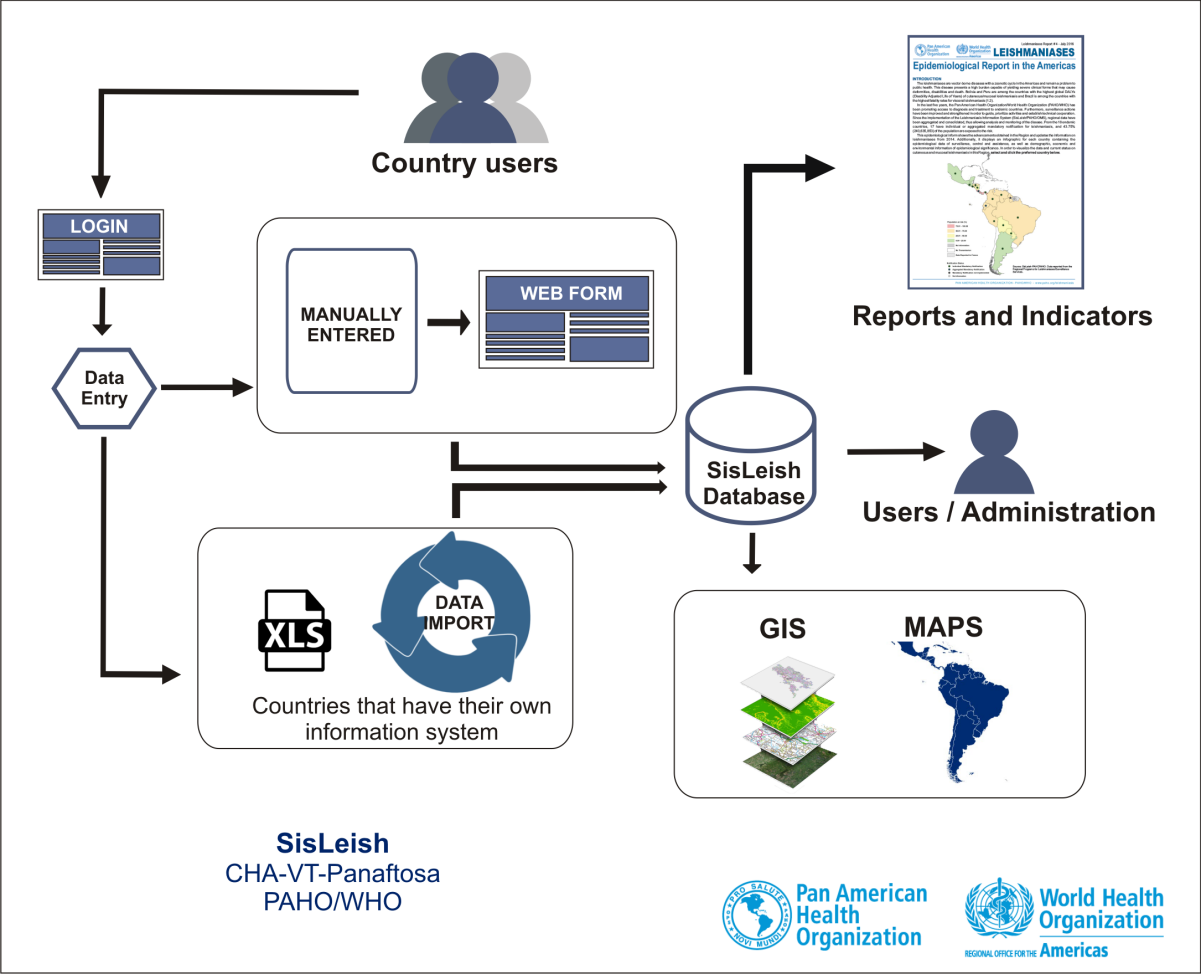
SisLeish data flow. Diagram representing the information data flow of SisLeish.

Annually, the RPL publishes the epidemiological report of Leishmaniasis in the Americas with the analyzed, standardized and compiled data from SisLeish for public viewing, available at the PAHO/WHO webpage <http://www.paho.org/leishmanisis>. The Data flow of SisLeish is represented by the diagram on Fig 1.

#### Cases definitions of leishmaniasis reported to SisLeish

The definition of “confirmed cases of leishmaniasis” was standardized by representatives from each Surveillance National Program as follows:

- Clinical Laboratory Criterion: suspected case of cutaneous, mucosal or visceral leishmaniasis with any laboratory-confirmed diagnosis.
- Clinical Epidemiology Criterion: suspected case of cutaneous, mucosal or visceral leishmaniasis, residing in an endemic area, without any laboratory-confirmed diagnosis, but with favorable therapeutic response to a specific treatment.

#### Descriptive variables

SisLeish possess different variables, which are grouped in three modules as described above, allowing data analysis per indicator and monitoring of the disease. The variables available in the system are detailed in Table 1.

**Table 1:** Description of the variables available in SisLeish.

Category	Variable
**General Data**	Year; Country; Division at the first and second sub-national administrative level; Total population at the second sub-national administrative level; Surface area (km^2^) of the second sub-national administrative level
**Demographic Data**	Gender; age group
**Clinical Data**	Clinical form; HIV status.
**Diagnosis confirmation criterion**	Laboratorial, clinical, epidemiological, unknown or uninformed.
**Types of Treatment**	Systemic, local, unknown or uninformed.
**Disease progression**	Recovery, death by leishmaniasis, death by other causes, unknown or uninformed.
**Etiological Agent and vector**	*Leishmania* species; *Lutzomyia* species.
**Input**	Surveillance, control and assistance module: Data on the Leishmaniasis National Program, vector and reservoir Surveillance and Control Program; Laboratories and Health Centers for Leishmaniasis; Medication availability.

Source: SisLeish-PAHO/WHO: Data reported from Regional Programs for Leishmaniasis/Surveillance Services.

#### Disease indicators

The indicators available in SisLeish were standardized and agreed upon by the endemic State Members of the region and are presented in the Table 2. Due to differences between reporting systems [14] the incidence denominator was standardized in order to allow comparison between countries, the current denominator is based on the total population from areas with transmission at the second administrative level.

**Table 2:** Description of the indicators available in SisLeish.

Indicators	Calculation	Purpose
**“Total number of cases” of leishmaniasis**	Total number of confirmed leishmaniasis cases that occurred in a given year, in the region, sub region or Country, at the first and second sub-national administrative levels; According to gender, age, clinical form (cutaneous, mucosal or visceral), HIV status, diagnosis criteria (laboratorial or clinical-epidemiological), type of treatment and disease progression.	Knowledge of the occurrence of leishmaniasis cases and its spatial and temporal distribution
**Case proportion**	Total number of confirmed leishmaniasis cases, according to gender, age, clinical form (cutaneous, mucosal or visceral), HIV status, diagnosis criteria (laboratorial or clinical-epidemiological), type of treatment and disease progression / divided by the “total number of leishmaniasis cases” that occurred in a given year in the region, sub-region or Country, at the first and second sub-national administrative levels X 100.	Knowledge of the magnitude of leishmaniasis cases in specific groups.
**Fatality rate**	Total number of deaths by leishmaniasis, according to clinical form / divided by the “total number of leishmaniasis cases” x 100	Knowledge of the leishmaniasis severity
**Incidence rate**	Total number of confirmed cases of leishmaniasis / divided by the total population at the second sub-national administrative level x 100.000 population	Knowledge of the magnitude and risk of occurrence
**Density rate**	Total number of confirmed leishmaniasis cases / divided by the surface area (km^2^) at the second sub-national administrative level x 1000	Knowledge of clusters in a geographic space
**Composite Indicator for Cutaneous Leishmaniasis (CICL)**	The composite indicator is product of the standardization and combination of the other three indicators (incidence rate, density rate and “total number of cases”) into one single metric. For this purpose the single scores were transformed into z-scores by subtracting the mean and dividing it by the standard deviation.	Knowledge of CL occurrence integrating the indicators (cases, incidence and density). The categories of the indicator are used to direct and prioritize surveillance, prevention and control actions in defined territories.
	$z=\frac{x-\mu }{\sigma }$	
	Where:	
	• ***x*** is the value of each single indicator (incidence rate, density rate and “total number of cases”) that we want to standardize as z-score. • ***μ*** is the mean of the population; • ***σ*** is the standard deviation of the population. • ***Z*** is the resulting z-score for each of these indicators, which is the absolute value that represents the distance between the raw score and the mean in units of the standard deviation; in short, how many standard deviations a value of a single indicator is from the population mean. • CICL = Σ Normalized case indicator + Normalized incidence indicator + Normalized density indicator • The CICL for each analyzed territorial unit was categorized by natural breaks, which allowed the generation of five strata of risk of transmission: low, moderate, high, intense and very intense.	

Source: SisLeish-PAHO/WHO: Data reported from Regional Programs for Leishmaniasis/Surveillance Services.

#### Geographic information systems

SisLeish utilizes geographic information systems to analyze the data, and produces maps as one of the outputs. To do so, the entire database from SisLeish were geocoded according to the standardized regional cartographic “basemap” from the Second Administrative Level Boundaries (SALB), created by the United Nations Geographic Information Working Group [15]. The cartographic base and country data have the same spatial unit and identical code (geocodes), which were included in SisLeish, allowing the automatic binding of the country data as attributes to the maps. Geographic Information System is subsequently utilized to map at the national, first and second sub-national level. The maps generated are available to the countries, so they can analyze their own data. The maps were created with the ArcGIS 10.2 software.

### Analyses of SisLeish

For the SisLeish analyses, we evaluated the system performance in aspects such as country adhesion and data completeness. Furthermore, we used the strengths-weaknesses-opportunities-threats (SWOT) technique, which is a simple tool to analyze different types of scenarios and support planning, management and improvement of projects, systems and companies [16]. This study utilized data available in the system from 2012 to 2015. Additionally, these analyses also describe the mayor ongoing improvements carried out since SisLeish was launched.

## Results

### Adhesion and data completeness

By 2015, all endemic countries have been granted access to SisLeish (Argentina, Bolivia, Brazil, Colombia, Costa Rica, Ecuador, El Salvador, Guatemala, Guyana, Honduras, Mexico, Nicaragua, Panama, Paraguay, Peru, Surinam, Uruguay and Venezuela).

**Fig 2:**
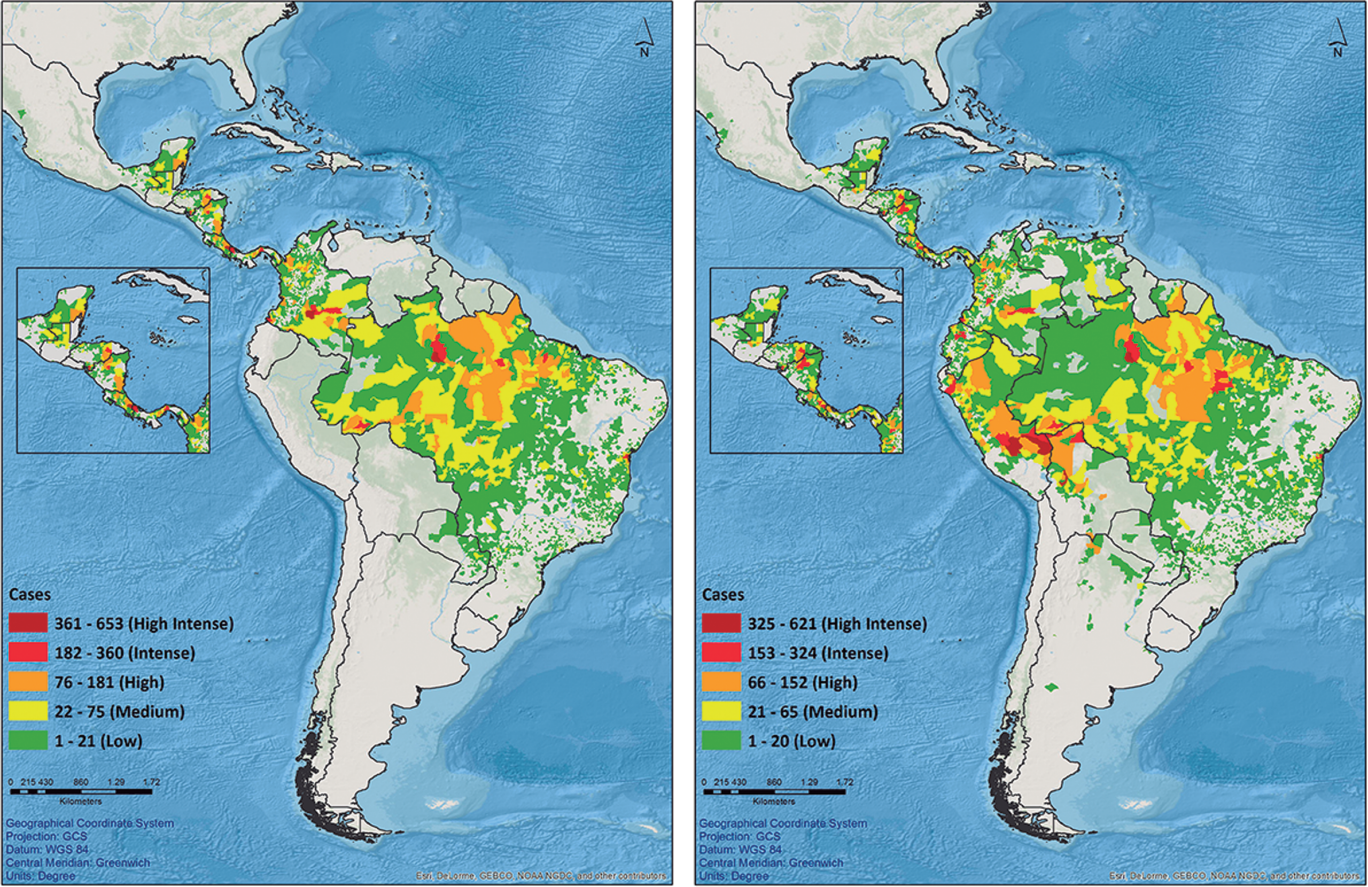
Map of adhesion to SisLeish. Maps displaying the adhesion of the countries at the second subnational administrative level for cutaneous/mucosal leishmaniasis from 2012 (map on the left) and 2015 (map on the right) in the Americas. Source. SisLeish-PAHO/WHO: Data reported from the Regional Programs for Leishmaniasis/Surveillance Services. Data available in February, 2017.

In 2012, six of the 12 countries with autochthonous VL reported data to SisLeish (2,892 cases), and 12 of the 18 countries with CL/ML transmission reported their cases (54,508). As for 2015, seven countries reported VL cases (3,456) and 17 reported CL/MCL cases (46,082). Further details of the progress on country adhesion can be observed in Table 3 and Fig 2.

**Table 3:** Adhesion to the system, number of first and second subnational administrative level units for cutaneous and mucosal leishmaniasis, and annual deadline from 2012 to 2015 in the Americas.

Adhesion and deadline	2012	2013	2014	2015
Number of countries that included CL/ML data	12	15	16	17
Number of countries that included CL/ML data at the second subnational administrative level	11*	14*	15*	16*
Number of first subnational administrative level units with CL/ML data	130	169	209	218
Number of second subnational administrative level units with CL/ML data	2,678	2,700	3,156	3,215
Number of countries that did meet the annual deadline for CL/ML case inclusion	3	6	6	10

*Guyana was not contained in this table seeing that this country does not have second subnational administrative level, only reporting at the first subnational administrative level.

Source. SisLeish-PAHO/WHO: Data reported from the Regional Programs for Leishmaniasis/Surveillance Services. Data available in February, 2017.

**Fig 3:**
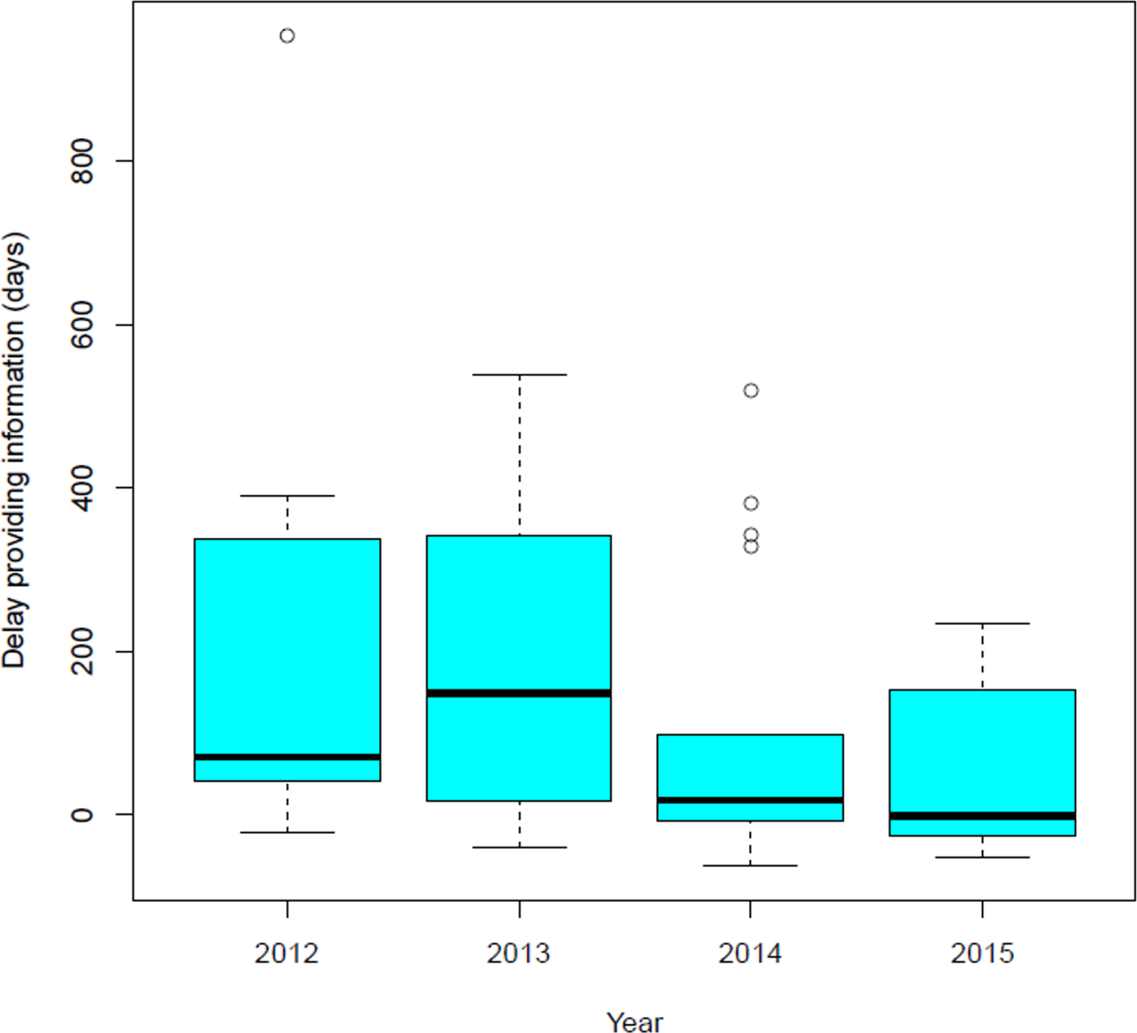
Delay in the insertion of data into SisLeish. Boxplots presenting the delay (days after the established date) in the insertion of the information into SisLeish by the countries per year. In this graph, the rectangle (box) reflects the values between quartile 1 and 3, which is where 50% of the central data of the distribution of results (time delay) is located. The widest line in the box is the median, which is the observation point that leaves 50% of the data below and 50% above. The "arms" above and below the box represent the maximum and minimum values. Outliers or extreme values (those values that are widely scattered compared to core data) are presented as points outside the arms.

Regarding the established deadline to enter the annual data (until April 30^th^ of each year), in 2012 only three (18%) countries did meet the deadline, increasing to 10 (59%) countries in 2015. The results are shown in Fig 3.

Concerning data completeness, there has been great progress for different variables present in the system since its implementation, such as gender, which had an approximately 100% improvement from 2012 to 2015. The progress of the system is shown in Table 4.

**Table 4:** Number and percentage of cases with information unknown from the total cutaneous/mucosal and visceral leishmaniasis cases of different variables in SisLeish, from 2012 to 2015.

	**Cutaneous and Mucosal Leishmaniasis**							
	**2012**		**2013**		**2014**		**2015**	
**Variables**	Number	%	Number	%	Number	%	Number	%
**Gender**	9,259	16.88	7,763	15.47	457	0.88	6	0.01
**Age group**	9,999	18.3	9,21	18.36	31	0.06	291	0.63
**Clinical Form**	3,374	6.15	4,222	8.42	4,442	8.63	837	1.82
**Diagnostic criteria**	10,554	19.26	13,688	27.29	62	12.04	3,691	8.01
**Type of treatment**	7,829	14.28	7,425	14.8	6,257	12.15	3,413	7.41
**Clinical progression**	34,704	63.28	30,588	60.98	27,603	53.61	22,798	49.48
**Total cases of CL/ML in the Americas**	54,508		50,163		51,492		46,082	
	**Visceral Leishmaniasis**							
	**2012**		**2013**		**2014**		**2015**	
**Variables**	Number	%	Number	%	Number	%	Number	%
**Gender**	0	0	3	0.1	0	0	0	0
**Age group**	20	0.69	29	1	39	1.34	21	0.6
**Diagnostic criteria**	0	0	16	0.47	15	0.41	0	0
**Clinical progression**	543	18.78	836	24.62	885	24.42	738	21.35
**Total cases of VL in the Americas**	2,892		3,396		3,624		3,456	

Source. SisLeish-PAHO/WHO: Data reported from the Regional Programs for Leishmaniasis/Surveillance Services. Data available in February, 2017.

### SWOT analysis of the system

**Fig 4:**
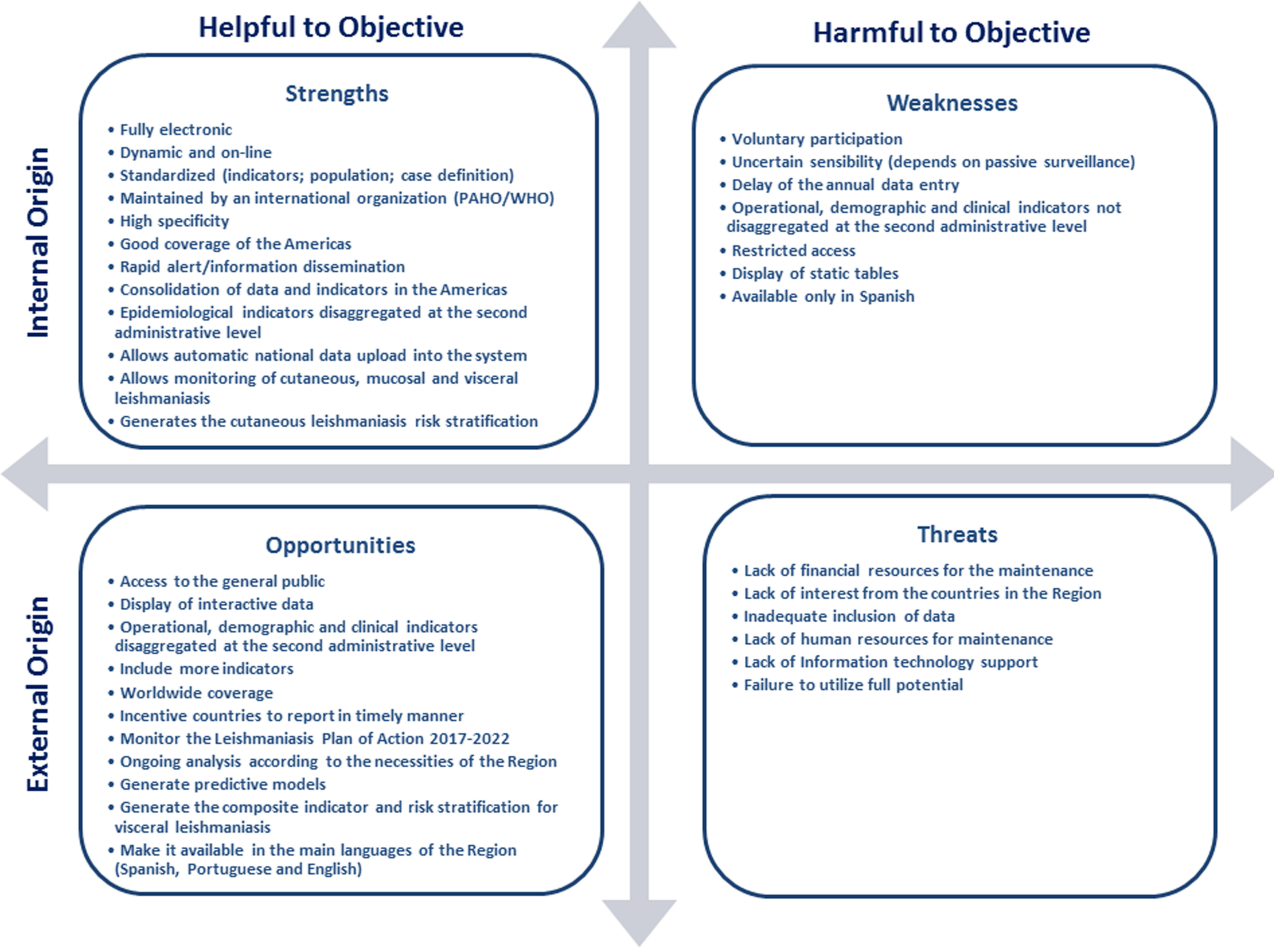
SWOT analysis. Summarized results of the SWOT analysis, outlining the most important, critical and relevant points for each internal and external factors.

The results of the SWOT (Strengths, Weaknesses, Opportunities and Threats) analysis are summarized in Fig 4 and it outlines the most important, critical and relevant points for each internal and external factors. The number of identified strengths (twelve) and opportunities (eleven) was greater than the weaknesses (seven) and threats (six), reflecting the importance of SisLeish for the leishmaniasis surveillance in the Americas, and also, highlighting the challenges that need to be addressed.

## Discussion

This study describes SisLeish, which is an information system that consolidates and analyses the leishmaniasis data, and allows monitoring and knowledge of the epidemiological status of 18 countries in the Americas. One of the main objectives behind the development of this system was to provide information to the countries and Regional Programs in order to guide surveillance and control actions, against CL/ML and VL, within the countries and to assist the definition of regional technical cooperation priorities.

In the Americas, leishmaniasis produces different clinical forms involving distinct species of *Leishmania* [2]. The confirmed cases of leishmaniasis reported to SisLeish were harmonized for the region, taking into account the definition of suspected cases for each country, in order to reduce biases and divergence between countries, this way permitting a uniform analysis of confirmed cases of leishmaniasis in the Americas.

There has been a considerable improvement in the adhesion, quality and data completeness. We observed a decrease in the delay to meet the established deadline to enter data into the system by the reduction of the median of days after the due date from 2012 (71 days after deadline) to 2015 (-1days after deadline). The results of SisLeish show the effort of the majority of the countries to improve their national database in terms of completeness and availability, mainly for CL/ML data, which is of compulsory notification in all of the endemic countries. In fact, SisLeish has stimulated several country programs to advance their surveillance system, and has been of particular incentive for countries that currently do not have a national information system available. For some countries, nevertheless, the notification of cases is aggregated at the second sub-national administrative level, which means a limitation in the quality of information provided by these countries. Thus, quantity and quality of information remain the main limiting factors of SisLeish, such as the well-known underreporting of leishmaniasis cases [7, 17, 18], and the high percentage of absence of clinical progression (recovery or death) data. Since this is, in general, a benign clinical form, many patients do not return to the health services for a follow-up and evaluation once the treatment is completed.

This information system has become an essential tool to improve knowledge and prioritize areas where the occurrence of leishmaniasis requires additional attention. The areas are identified utilizing the information provided by three single indicators, on the occurrence of leishmaniasis, and the results of the CICL. The solely use of single indicators (e.g. incidence, density or the total cases) could lead, however, to an incomplete, or even misleading, interpretation of the occurrence. For instance, the relevance of an area with high number of cases could be “diluted” when accounting for the population and/or surface area, potentially deflecting the real problem. In contrast, the CICL allows combining the different indicators of occurrence, presenting their overall standardized additive value. Additionally, the Jenks natural breaks classification method allowed SisLeish to provide meaningful results on how to split the different categories of the CICL, in “natural clusters”. Thus, for example, SisLeish is able to identify areas with particularly very high results in the CICL, compared with the other categories, which are denoted as “very intense transmission”. These groups are clearly differentiated areas, since the Jenks method seeks to reduce the variance within classes while maximizing the variance between them [19]. Other widely used methods for splitting categories, such “quartiles” or “equal intervals”, would not provide similar useful results, as it can be argued that these methods split the data rather arbitrarily, regardless of the data distribution. Therefore, the CICL categorization contributes better to the identification of an actual need to prioritize actions and resources for the surveillance and control of this disease.

When choosing health indicators it is essential to understand the nature of the data and the purpose behind the development of an indicator, otherwise the indicators could actually confuse or misguide decision-makers [20]. In SisLeish, the development process of the CICL was accompanied by experts and representatives of the Leishmaniasis Programs from endemic countries. The outputs were assessed to determine its performance, as well as the extent of usefulness, and the new CICL demonstrated to be sensitive to highlight areas where the incidence, density or “total number of cases” were important.

Despite knowing the limitations of the use of single indicators for decision-making processes, the composite indicator for VL is still unavailable in the system. Brazil represents 96% of the VL cases in the Americas, and since 2003 the Brazilian MoH has been stratifying the areas of transmission, based exclusively on the average of human cases from the previous three years, to direct the surveillance and control actions. In addition to the limitation of the “average of cases” indicator, there is also a need to include other relevant epidemiological data, such as vector, infected dogs, among others; however, these additional data are not fully available and do not hold the required quality to be incorporated in an epidemiological analyses. In this context, due to a request from the Brazilian MoH, the RPL is currently working to combine the data available in the system into another composite indicator for VL.

The visualization of the categorized data and indicators in maps allows a better understanding of the leishmaniasis epidemiological status. The database geocoding of SisLeish, at the first and second sub-national administrative levels, allows systematic mapping, which enables geographical monitoring of the occurrence. Hence, the data should be available at the most disaggregated administrative level to facilitate local analyses that contribute to the guidance and effectiveness of surveillance and control actions [21, 22]. The information from SisLeish has shown an increase in the availability of disaggregated data of the endemic countries, at the second sub-national administrative level, from 64.7% in 2012 to 94% in 2015. However, given that the SALB cartographic database incorporated into SisLeish was last updated in 2012, administrative divisions modified or created after this event might be misrepresented in the output maps, demanding the need for a more up-to-date cartographic database.

Through the use of geographical information systems tools [23, 24, 25, 26], SisLeish contributes to the knowledge of the geographical distribution of the disease in different scales. Additionally, the system can assist the development of other analyses in the future, by incorporating socioeconomic, distribution of the human population and environmental data, which could lead to the better understanding of the determinants of this disease. Hence, it would be possible to analyze particular determinants and circumstances capable of inducing the development of leishmaniasis in areas of epidemiological importance, allowing more adequate planning of actions aimed at prevention, surveillance and control of this disease.

Acceptance and implementation of an information system is one of the greatest challenges for epidemiological surveillance programs, which require continuous monitoring to reinforce the countries commitment to share information. Furthermore, SisLeish is providing support to sub-regional projects for cross-border surveillance, facilitating the exchange of information on the occurrence of leishmaniasis in the country borders. Additionally, SisLeish has developed a pilot alert system for VL in the borders of the MERCOSUR countries, which allows data sharing on phlebotomine presence, human and canine VL cases. Likewise, a surveillance, control and assistance module was created in 2015 to monitor and follow up with National Programs; this module was designed to become compatible with the database from the WHO, in order to reduce efforts and to avoid database duplicity. At first, this module had a low adhesion, only incorporating the data from two countries; nonetheless, there was a rise to nine countries in 2015.

Despite being a simple methodology, the SWOT analysis has been increasingly used by managers and professionals to identify the strengths, weaknesses, opportunities and threats of programs, services or systems, to define and select appropriate strategies to improve actions and goals [27, 28]. Fig 4 summarizes the SWOT analysis of SisLeish, showing that the strengths and opportunities exceed the initial expectative of the internal and external factors of the origin analysis, which are helpful to the objectives of the system. Nevertheless, the identified weakness and threats represent the frailties of the system, showing its limitations and calling attention to the needs of the system and what must be improved.

Currently, SisLeish is undergoing a restructuring to enhance its functionality, which implies an improvement of the graphic and geographic interfaces, with the addition of interactive reports and maps, and providing public access to its information. Likewise, the system is being translated into the three main regional languages (i.e. Spanish, English and Portuguese); given that nowadays it is only available in Spanish.

Since the implementation of SisLeish, the adhesion is continually growing, and presently has the data from all endemic countries available in the system. Therefore, it is imperative to continue encouraging countries to report their data, through improvements and an ongoing evolution of the system, so it can remain as an important surveillance tool in order to guide and implement actions.
